# Broad diversity of *Mycobacterium tuberculosis* complex strains isolated from humans and cattle in Northern Algeria suggests a zoonotic transmission cycle

**DOI:** 10.1371/journal.pntd.0008894

**Published:** 2020-11-30

**Authors:** Hanane Damene, Djamel Tahir, Maren Diels, Ali Berber, Naima Sahraoui, Leen Rigouts

**Affiliations:** 1 Institute of Veterinary Sciences, University Blida 1, Blida, Algeria; 2 IHU Méditerranée Infection, Marseille, France; 3 BCCM/ITM Mycobacterial Culture collection, Institute of Tropical Medicine, Antwerp, Belgium; 4 Mycobacteriology Unit, Institute of Tropical Medicine, Antwerp, Belgium; 5 Department of Biomedical Sciences, University of Antwerp, Antwerp, Belgium; KU Leuven, BELGIUM

## Abstract

*Mycobacterium tuberculosis* complex (MTBC) comprises closely related species responsible for human and animal tuberculosis (TB). Efficient species determination is useful for epidemiological purposes, especially for the elucidation of the zoonotic contribution. In Algeria, data on MTBC genotypes are largely unknown. In this study, we aimed to investigate the occurrence and diversity of MTBC genotypes causing human and bovine TB in Northern Algeria. During a two-year sampling period (2017–2019) in two regions of Northern Algeria, we observed an overall prevalence of 6.5% of tuberculosis (TB) among slaughtered cattle, which is higher than previous Algerian data yet comparable to neighboring countries. A total of 296 *Mycobacterium tuberculosis* complex (MTBC) isolates were genotyped by spoligotyping: 181 from tissues with TB-like lesions collected from 181 cattle carcasses and 115 from TB patients. In human isolates, we identified 107 *M*. *tuberculosis*, seven *M*. *bovis* and one “*M*. *pinnipedii-like*”, while for bovine samples, 174 isolates were identified as *M*. *bovis*, three as *M*. *caprae*, three as “*M*. *pinnipedii-like*” and one as “*M*. *microti-like*”. The majority of isolates (89.2%) belonged to 72 different known Shared International Types (SIT) or *M*. *bovis* spoligotypes (SB), while we also identified seven new SB profiles (SB2695 to SB2701). Twenty-eight of the SB profiles were new to Algeria. Our data suggest zoonotic transmission in Sétif, where significantly more TB was observed among cattle (20%) compared to the slaughterhouses from the three other regions (5.4%–7.3%) (p < 0.0001), with the isolation of the same *M*. *bovis* genotypes from TB patients. The present study showed a high genetic diversity of MTBC isolated from human and cattle in Northern Algeria. Even though relatively small in terms of numbers, our data suggest the zoonotic transmission of TB from cattle to humans, suggesting the need for stronger eradication strategies for bovine TB.

## Author summary

To establish the circulating genotypes causing human and bovine tuberculosis (TB) in Northern Algeria, spoligotyping was used to characterize 296 *Mycobacterium tuberculosis* complex isolates of human (n = 115) and bovine (n = 181) origin. Molecular identification showed that most human isolates were identified as *M*. *tuberculosis*, with few *M*. *bovis* isolates, while among cattle, *M*. *bovis*, *M*. *caprae*, “*M*. *pinnipedii-like*” and “*M*. *microti-like*” isolates were found. Among *M*. *tuberculosis* isolates, 84 (78.5%) were classified into six families, with three prevailing genotypes (Latin–American Mediterranean (LAM), T clade and Haarlem). Concerning *M*. *bovis*, three spoligotype profiles (SB0120, SB0121 and SB0134) were predominant in Algeria, as in other Northern African countries. In addition, we identified some new *M*. *bovis* types (SB2695 to SB2701). For the first time in Algeria, *M*. *bovis* was found in 6% of human cases analyzed in this study. Furthermore, we documented regional variability in the prevalence of bovine TB among slaughtered cattle and in the probability of human TB being caused by *M*. *bovis*, suggesting the zoonotic transmission of *M*. *bovis*. This finding should draw the attention of Algerian health authorities to the importance of the effective control of bovine TB, including the establishment of traceability systems for livestock from the farm to slaughterhouse and between farms.

## Introduction

Among infectious diseases, tuberculosis (TB) is one of the most significant in terms of public and animal health because of its high morbidity and mortality rate in humans, in addition to the economic losses related to affected herds [1,2]. The infection is caused by bacilli of the *Mycobacterium tuberculosis* complex (MTBC) [3], which includes 11 (sub-) species: *M*. *tuberculosis*, *M*. *bovis*, *M*. *africanum*, *M*. *microti*, *M*. *canettii*, *M*. *caprae*, *M*. *pinnipedii*, *M*. *suricattae*, *M*. *mungi*, *M*. *orygis* and the dassie bacillus. From those, *M*. *tuberculosis* is the most prevalent causative agent of human pulmonary TB [4], infecting more than one-quarter of the world’s human population [5]. *M*. *tuberculosis* can occasionally infect animals (*e*.*g*., birds, elephants, and other mammals) that have prolonged and close contact with humans [6,7]. According to the World Health Organization (WHO), more than 10 million human TB cases were reported in 2018. The largest incidence rate of human TB occurred in the South-East Asian region, followed by the African region and the Western Pacific, with 44%, 24% and 18% of new cases, respectively [8].

*M*. *bovis* is the main causal agent of animal and bovine TB and can cause zoonotic TB in humans through ingestion (*e*.*g*., the consumption of unpasteurized milk), inhalation and—less frequently—by contact with mucous membranes and broken skin [9]. Human infection by *M*. *bovis* is often associated with extra-pulmonary disease [10]. Data on the prevalence of *M*. *bovis* in human infection are scarce but generally higher in low-income regions, with a high prevalence in cattle [11,12]. About 70,000 cases are estimated to occur annually in Africa [13].

Algeria is among the countries in which human and bovine TB are endemic. In 2018, more than 23,000 cases of human TB were reported, with a high incidence of extra-pulmonary cases (69.43%) [14]. This unexpected high level of reported extra-pulmonary TB may be linked to the applied criteria for diagnosis, which are mostly based on (para)clinical (tuberculin skin test, imaging and histopathology) rather than bacteriological findings (personal communication, National Reference Laboratory, Algiers). In general, TB laboratory diagnosis is limited to conventional tools such as microscopic examination using the Ziehl–Neelsen (ZN) technique and sample culture on Löwenstein–Jensen (L-J) medium. Concerning bovine TB, despite the efforts made by the Algerian authority to control the disease by setting up a national screening program based on tuberculin skin testing and the elimination of all positive cattle, combined with abattoir meat inspection and the condemnation of affected carcasses, bovine TB remains prevalent (3.6%) [15,16].

Over the last few years, the development of methods for the molecular epidemiology of TB has helped to create a better understanding of this disease and its causative agent, MTBC [17]. Among these, spoligotyping is currently one of the most widespread. This simple PCR-based approach is useful for studying the phylogeography of MTBC organisms from isolates or clinical specimens. It relies on the polymorphism at one particular genomic region: the so-called direct repeat (DR) locus [18]. Polymorphisms among isolates represent the presence or absence of spacer sequences separating the direct repeats [19]. Results are represented as a binary code or an octal designation and are analyzed with web tool databases, where strains can be assigned to a specific clade and situated in a phylogenetic tree [20]. Spoligotyping has proven to be a practical and discriminatory method for large-scale studies of the epidemiology of *M*. *bovis* as well as for the differentiation of *M*. *bovis* from *M*. *tuberculosis* [21].

In Algeria, data on MTBC genotypes implicated in human and bovine TB are scarce or non-existent. The objectives of this study were to conduct a molecular characterization of both human and bovine MTBC in two departments of Northern Algeria using spoligotyping and to identify the potential exchange of strains with other countries as well as between cattle and humans.

## Materials and methods

### Ethics statement

The study protocol for human subjects was reviewed and approved by the Ethics committee of the University of Sétif (Ref. Number 015/CED/2019). Obtaining informed consent was not required for this study because individual patient data (age and sex) have been rendered and reported anonymous so that the patient is no longer identifiable. The authorization to conduct the field study in slaughterhouses (Ref. Number 449/IVW/17) was obtained from the Inspection Vétérinaire de Wilaya, operating under the auspices of the Direction des Services Vétérinaires (DSV, Ministry of Agriculture).

### Study setting

The present study was conducted in two departments (wilayates) in Algeria: Béjaia is located in the North (36° 45' 00" N, 5° 04' 00" E), and Sétif, the capital of the highlands, is in North-Eastern Algeria (36° 09' 00" N, 5° 26' 00" E). According to the latest census (2018), Béjaia and Sétif have populations of 978,050 and 1,489,495 inhabitants, respectively. The departments are served by eight and seven diagnostic centers for human TB and respiratory disease control, respectively. Human samples were collected from two of these TB diagnostic centers (Service de Contrôle de la Tuberculose et des Maladies Respiratoires; SCTMR), with one each in Béjaia and Sétif.

In general, the Northern region of the country is known for its agricultural activity, including cattle breeding of different categories: local breeds (48%), cross-breeds (42%) and imported breeds (10%). Béjaia is known for its dairy cattle and goat farms, while cattle and sheep farms are concentrated in Sétif. Four municipal slaughterhouses were included in this study: Béjaia center and Kherrata from the Béjaia department, and Sétif center and El-Eulma from the Sétif department.

Samples were collected over a period of two years, from November 2017 to November 2019, with the exception of human sampling in Sétif, which was limited to a one-year period from November 2017 to December 2018.

### Sample collection

The four included slaughterhouses were each visited twice a week throughout the study period. All bovine carcasses subjected to sanitary inspection that presented macroscopic lesions such as tubercles or foci with different types (caseous or calcified), single or multifocal, circumscribed or poorly delimited, were considered as TB-like lesions. All these carcasses were included in the present study, making an average of about 10 cattle per day per abattoir. Tissue samples were collected from typical tubercles or lymph nodes with TB-like lesions and stored individually in sterile, labeled containers. All collected samples were transported to the SCTMR of Béjaia and stored at -20°C for later batched bacteriological analyses. Information related to the identification of each animal (age, sex and breed) and tissue sample (organ/tissue affected and description of lesion) was recorded in our database.

For human TB, we exploited the isolates collected from the two SCTMRs located in Béjaia and Sétif. These isolates were obtained from a total of 1952 presumptive TB patients submitted to routine TB diagnostic procedures. Demographic data (age and sex) and laboratory outcomes (ZN staining, culture and type of sample) were reviewed from medical records.

### Tissue preparation and bacteriological analyses

All animal tissue samples were homogenized before bacteriological examination. Approximately 5g of each specimen was cut in a sterile Petri dish using sterile blades and forceps to obtain fine pieces and further homogenized using a sterile mortar and pestle. The obtained ground tissue was decontaminated by the modified Petroff method [22]. The sediment of decontaminated samples was used for microscopic examination by ZN staining and culture by inoculation on Coletsos and L–J media. Culture tubes were incubated at 37°C for 8 to 12 weeks, with weekly observation for the growth of colonies. The presence of acid-fast bacilli (AFB) was checked by ZN staining. In accordance with routine practice, human samples underwent microscopic examination by ZN staining and culture on L–J medium.

### Spoligotyping and phylogenetic analysis

For the genotyping of all isolates, a loop full of each positive culture was harvested in a sterile Eppendorf tube and suspended in 0.5 ml of sterile distilled water. All isolates were then inactivated by incubation at 100°C for 5 min in a boiling water bath and stored at -20°C until their shipment to the Institute of Tropical Medicine in Antwerp, Belgium. For each isolate, 4 μl of the denatured bacterial suspensions were used for spoligotyping following Kamerbeek’s protocol [21]. The *M*. *tuberculosis* (H37Rv, ITM 2008-03715) and *M*. *bovis* BCG (ITM 1998–00269) reference strains were used as positive controls, while distilled water was used as a negative control in each run. The analysis of spoligotyping data and construction of the dendrogram were conducted by using the MIRU-VNTR*plus* international database (http://www.miru-vntrplus.org), while the designations of international names for spoligotype profiles of bovine origin were obtained from the *Mycobacterium bovis* spoligotype database (http://www.M.bovis.org/). For a subset of isolates that were not closely grouped to the typical *M*. *bovis* or *M*. *tuberculosis* spoligotypes, we also analyzed lineage-specific single-nucleotide polymorphisms (SNPs) and the MTBC regions of difference (RDs).

### PhyloSNP

The PhyloSNP analysis is based on the identification of lineage-specific SNPs [23]. We used optimized settings which differed slightly from those used by Cancino-Muñoz (S1 Text). The test consisted of two PCRs, one targeting SNPs specific for lineages 1, 2 and 5 (L1–L2–L5) and the other for L3–L4–L6, followed by Sanger sequencing (outsourced to Baseclear, The Netherlands). The sequences were aligned to the H37Rv *M*. *tuberculosis* reference strain using CLC sequence viewer 8 and SnapGene viewer 4.2.9 (for visualization) to identify lineage-specific SNPs as well as double peaks indicating mixed infections, if any were present.

### Multiplex RD-PCR

We applied the two-step, multiplex PCR method based on genomic RDs (RD1, RD1mic, RD2seal, RD4, RD9 and RD12) for the differentiation of *M*. *tuberculosis*, *M*. *africanum*, *M*. *bovis* (BCG), *M*. *caprae*, *M*. *microti*, *M*. *pinnipedii* and *M*. *canettii* as described by Warren and colleagues [24].

### Data analysis

To calculate the p-values and odds ratio, we used MedCalc freeware (https://www.medcalc.org/calc/odds_ratio.php; accessed 06/07/2020).

## Results

### Clinical data of cattle samples

**Fig 1:**
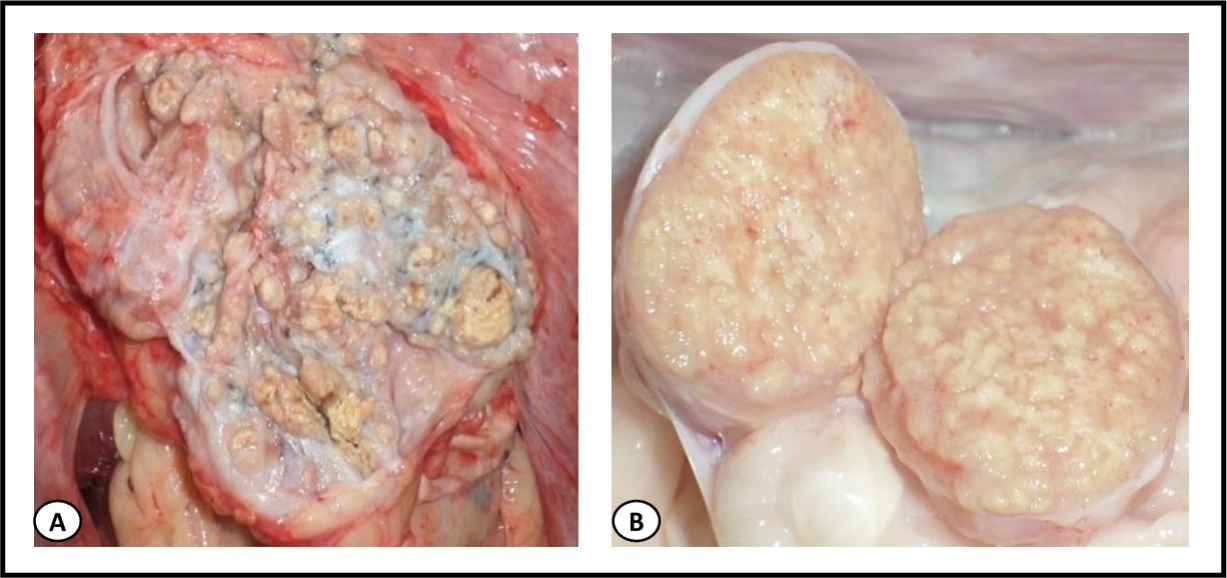
Granulomatous alterations of pulmonary lymph nodes. (**A)** Multiple small circumscribed tubercles with yellow content in the left tracheobronchial lymph node in a cow aged more than 5 years. (**B)** Transverse section showing several white-yellowish, irregular foci in the medial mediastinal lymph node in a calf aged <2 years.

**Fig 2:**
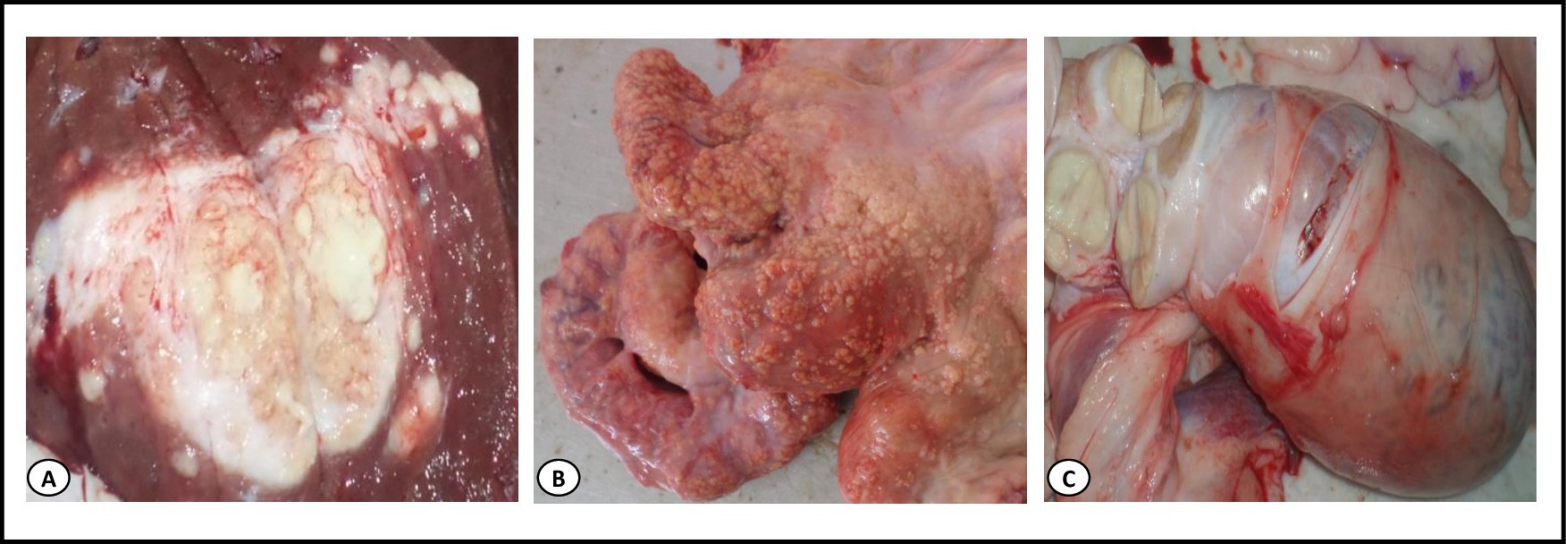
Suspected lesions of bovine tuberculosis in extra-pulmonary organs. **(A)** A large focus of yellow caseating necrosis in the liver tissue. (**B)** Numerous small, grey to white-yellowish, confluent tubercles in the uterus (miliary tuberculosis). (**C)** Round tuberculous granuloma containing a yellow, dry caseous necrosis central, surrounded by a thin brown zone and an outer broader, white-grey zone in the testis lymph node.

**Fig 3:**
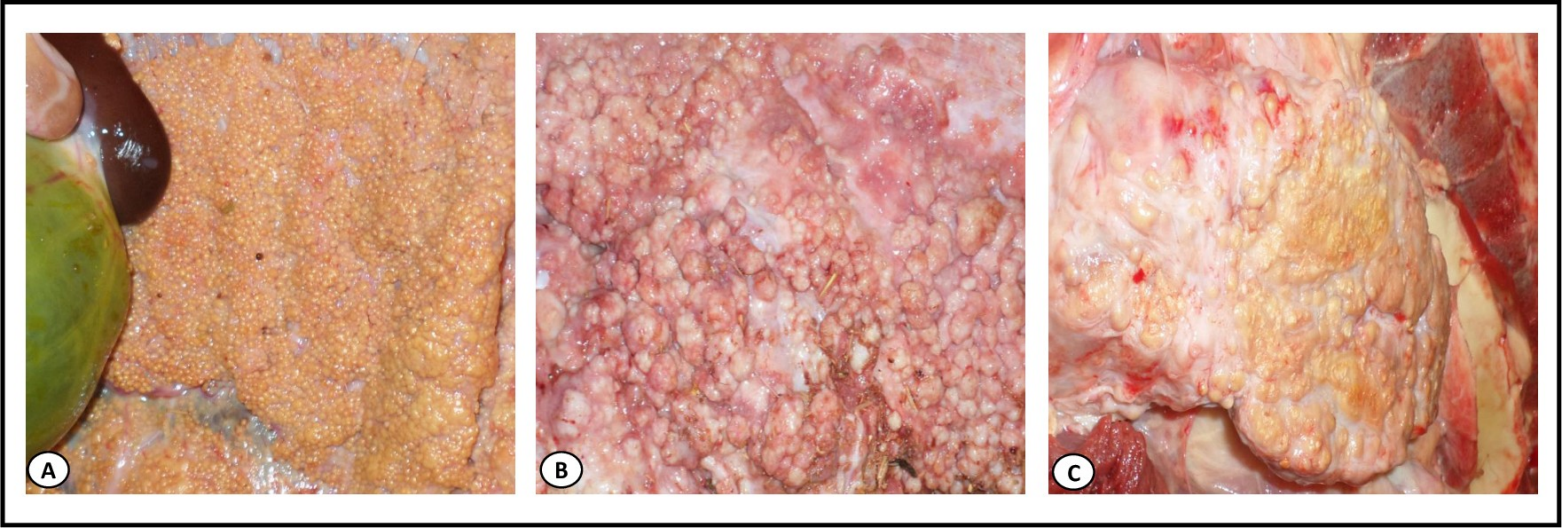
Generalized tuberculosis lesions in different bovine organs. **(A)** Small tubercles that are grey to white-yellowish on parietal pleura (miliary disease). (**B)** Multiple tubercles that resemble pearls of a gray-reddish color in the lung tissue (pearl disease). (**C)** Large caseous mass in the lung tissue.

A total of 3546 cattle carcasses were subjected to sanitary inspection for the detection of TB-like lesions during the study period. *Post-mortem* inspection showed an overall prevalence of carcasses with TB-like lesions of 6.5% (232/3,546), with inter-region variability representing 5.4% (35/650) of cattle in Kherrata, 6.1% (150/2,460) in Béjaia and 7.3% (23/316) in El-Eulma abattoirs, which is significantly less compared to the 20% (24/120) in Sétif (p < 0.0001; Table 1). The distribution of lesions in organs showed that tubercles were most often (66.8%) localized in pulmonary lymph nodes, while 28.9% of cattle had lesions in both lungs and extra-pulmonary organs (liver, head, heart, abdominal and thoracic cavities, thymus, female and male genitalia, etc.) (Table 2 and Figs 1 and 2) and 4.3% of infected cattle had generalized TB (Table 2 and Fig 3).

**Table 1:** Repartition of prevalence of bovine TB-like lesions in four abattoirs in Northern Algeria from November 2017 to November 2019.

Slaughterhouses	No. of inspected cattle	No. (%) of carcasses presenting suspected lesions of bovine TB	No. (%) of culture positive samples
**Béjaia**	2,460	150 (6.1)	122 (81.3)
**Kherrata**	650	35 (5.4)	17 (48.6)
**El-Eulma**	316	23 (7.3)	23 (100)
**Sétif**	120	24 (20) *	19 (79.2)
**Total**	**3,546**	**232**	**181**

* OR = 3.87 (95% CI 2.42–6.18); p<0.0001.

**Table 2:** Summary of repartition of bovine TB-like lesions in various organs.

Tissues/organs		No. of carcasses with lesions
**Pulmonary (LN) /Tissue**		155
**Pulmonary and extra-pulmonary**	Lungs + liver (LN)	27
	Lungs and/or liver + head (LN)	14
	Lungs and/or liver + mesenteric (LN)	6
	Lungs + pre-scapular or pre-crural (LN)	6
	Lungs (LN) + thoracic cavity	4
	Lungs and/or manibrial + head + liver + mesenteric (LN)	3
	Lungs and/or mesenteric + head + pre-crural + manibrial (LN)	2
	Lungs and/or testis + liver + precrural (LN)	2
	Lungs + head+ prescrapular + mesenteric (LN)	1
	Lungs (LN) + heart	1
	Lungs + manibrial (LN) + thoracic cavity + thymus	1
**Generalized tuberculosis**		10

**Fig 4:**
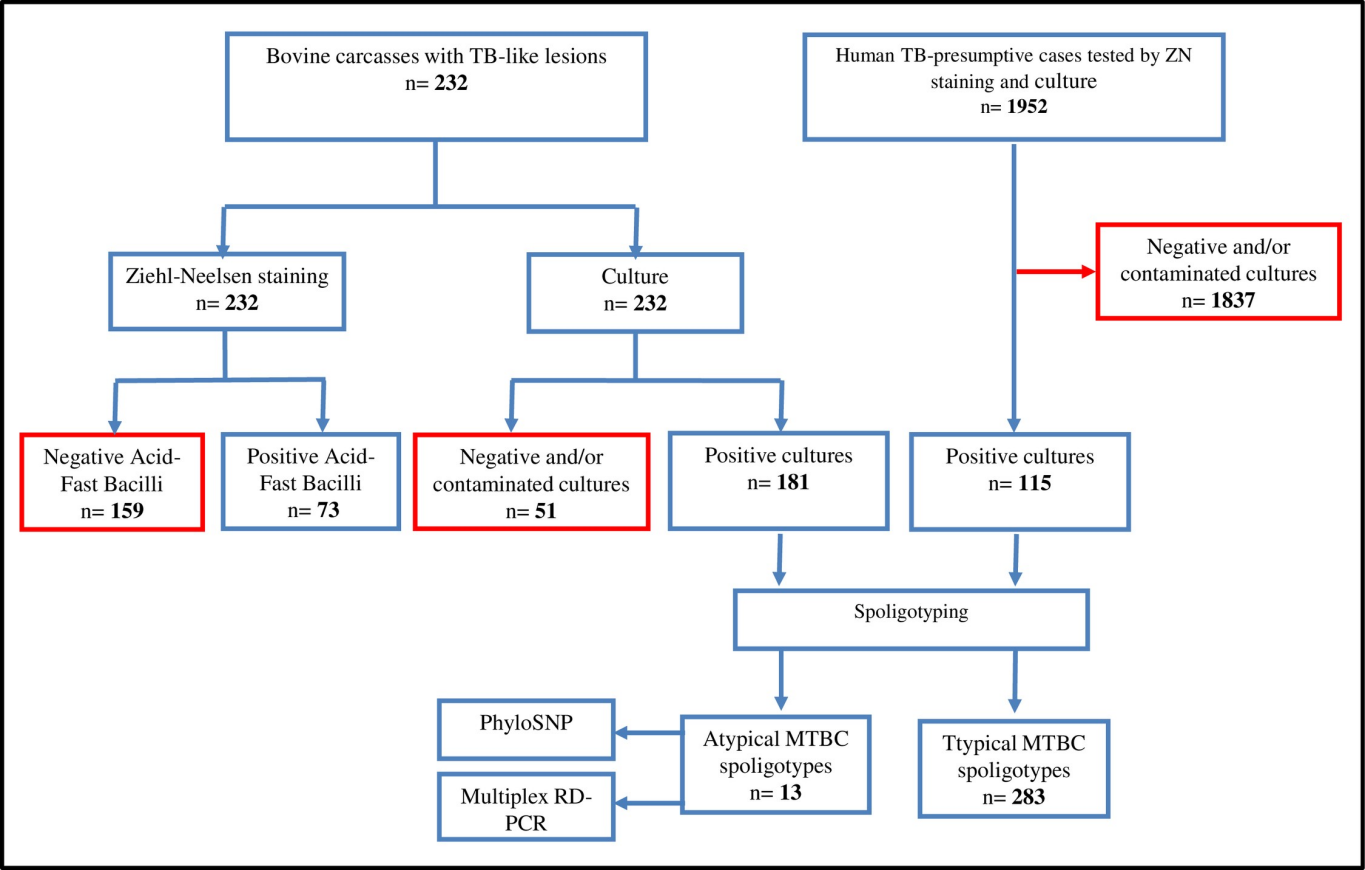
Schematic representation of the human and bovine sample analysis strategy.

Out of the 232 tissue samples, 73 (31.5%) were AFB-positive by direct microscopy. Further, bacterial growth was obtained for 181 samples (78%) (Fig 4), from which 64 were AFB-positive by direct smear microscopy examination. We did not observe a difference in final positivity on Coletsos versus L–J medium.

### Human samples and clinical isolates

In humans, a total of 115 cases were confirmed as being MTBC by culture (Fig 4), with 87 cases originating from the SCTMR of Béjaia (2-year sampling) and 28 from Sétif (1-year sampling). Among the 115 confirmed TB cases, 83 (72.2%) were isolated from specimens associated with pulmonary disease (51 sputum, seven bronchial aspiration fluids and 25 gastric aspirations) and 32 (27.8%) from extra-pulmonary specimens (18 pleural biopsies, one bowel biopsy, nine pleural fluids, two pus and two cerebrospinal fluid). Gastric aspirates were obtained from hospitalized adults with three smear-negative sputum specimens. Sixty-five patients were males (sex-ratio 1.3) with an age range of 18–97 years (Table 3).

**Table 3:** Demographics and clinical data characteristics of TB patients.

Parameters		No. (%) of TB patients
Sex		
Male		65 (56.5)
Female		50 (43.5)
Age group		
< 20 years		3 (2.6)
21–60 years		60 (52.2)
> 60 years		24 (20.9)
Not recorded		28 (24.3)
Smear microscopy		
Pulmonary form	Positive	24 (28.9)
	Negative	59 (71.1)
Extra-pulmonary form	Positive	1 (3.1)
	Negative	31 (96.9)

### Spoligotyping analysis

Spoligotyping confirmed MTBC species for all isolates, identifying a total of 181 *M*. *bovis*, 107 *M*. *tuberculosis*, three *M*. *caprae*, four close to the seal profile (“*M*. *pinnipedii-like*”) and one close to the vole profile (“*M*. *microti-like*”) (S1 Fig). Of the 115 human isolates, 107 were identified as *M*. *tuberculosis*, seven as *M*. *bovis* and one “*M*. *pinnipedii-like*”. No *M*. *tuberculosis* was identified in cattle. The *M*. *caprae*, “*M*. *microti-like*” and three remaining “*M*. *pinnipedii-like*” isolates originated from cattle.

In total, 97 distinct spoligotype patterns were identified, with 235/296 (79.4%) of isolates being grouped into 36 clusters containing 2–61 isolates. Among the cattle isolates, a total of 42 different spoligotypes were identified, with 155/181 (85.6%) of isolates being grouped into 16 clusters containing 2–57 isolates (S1 Fig).

Overall, 264 (89.2%) isolates could be assigned to 72 different published Shared International Types (SIT) or *M*. *bovis* spoligotypes (SB), while 24 (8.1%) isolates could not be assigned an SIT/SB (considered “orphans”) and eight cattle isolates were assigned to seven new SB profiles (SB2695 to SB2701) (Figs S1 and 5). Two of these new profiles (SB2695 and SB2696) differed by only one spacer from previously described *M*. *bovis* spoligotypes (SB0134 and SB1874, respectively), while two others (SB2697 and SB2698) matched the “typical *M*. *bovis* profiles” despite multiple spacer differences (S1 Fig). The remaining three new profiles were more distinct (S1 Fig).

The SB2699 isolate from Sétif was grouped with two isolates from Béjaia showing the SB2404 profile previously described in France (S1 Fig). These three cattle isolates were most closely related to an “orphan” human isolate from Sétif and not far from the cattle isolate from El-Eulma with the new SB2701 profile, which in turn was closest to the “seal” profile (*M*. *pinnipedii*). The SB2701 strain was isolated from an adult (three-years-old) male bovine with miliary TB (S2 Fig). RD-PCR analysis showed the typical “*M*. *bovis*” RDs profile with an absence of RD4, RD9 and RD12 for SB2699 and SB2404, although an uninterpretable result was found for RD4 in the case of the SB2701 isolate. PhyloSNP analysis did not detect any of the SNPs specific for *M*. *tuberculosis* lineages L1 to L6 for any of these isolates. Despite the suggested close link to the “seal” profile based on spoligotyping, the SB2701 isolate exhibited an RD2seal band, which is typically missing from *M*. *pinnipedii*.

The last new profile (SB2700) most closely matched the “vole” profile (*M*. *microti*) (S1 Fig). It was isolated from the caseous-calcified lesion of the right tracheobronchial lymph node of an adult (two-year-old) male bovine (S2 Fig). Despite the suggested close link to the “vole” profile based on spoligotyping, the SB2700 isolate exhibited the RD1mic band, which is typically missing from *M*. *microti*. RD9 and RD12 were missing, suggesting classification as *M*. *bovis*, but no band was obtained with the RD4 primers, which is not in agreement with the *M*. *bovis* classification. PhyloSNP analysis did not detect any of the SNPs specific for *M*. *tuberculosis* lineages L1 to L6.

Twenty-eight of the spoligotypes from bovine origin, representing 23.7% (43/181) of our cattle isolates, were not yet reported in Algeria (S1 Table). With the exception of five existing SB types and the seven new SB types, all SB types have been reported before in neighboring Northern African or Southern European countries (S1 Table). Most of the isolates belonging to SB types previously reported in Algeria were part of a cluster in this study (S1 Table). The highest frequency was recorded for SB0120 (n = 57, 31.5%) followed by SB0121 (n = 41, 22.6%) and SB0134 (n = 18, 9.9%). The three *M*. *caprae* isolates belonged to SB0835 (two isolates; one from Béjaia and one from Sétif) and SB1451 (one isolate from Sétif) spoligotypes.

**Fig 5:**
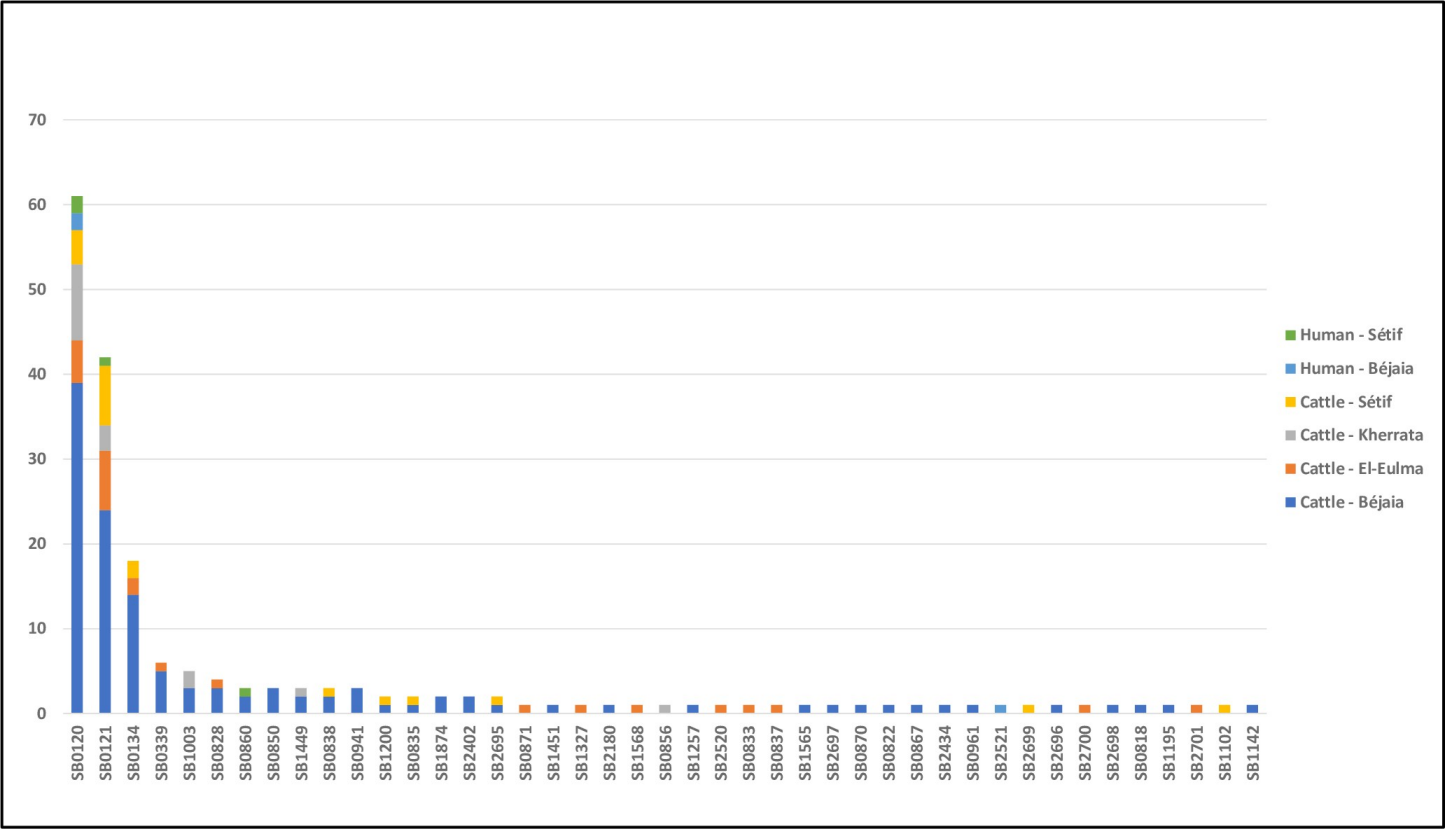
Distribution of *M*. *bovis* spoligotypes among bovine and human samples from two departments in Algeria (2017–2019).

The human *M*. *bovis* isolates (n = 7) were assigned to four different spoligotypes, of which six were grouped with bovine isolates (SB0120, SB0121, SB0860), while one isolate (SB2521) did not match any of our cattle isolates (S1 Table and Fig 5). It is worthy of note that SB2521 differs in only one spacer from the more common SB0134. The proportion of *M*. *bovis* isolation from patients with extra-pulmonary TB (2/32, 6.2%) did not differ from that in pulmonary TB patients (5/83, 6%) (p = 0.9638; S2 Table). *M*. *bovis* represented 14.3% (4/28) of human MTBC isolates from Sétif and 3.4% (3/87) of Béjaia human isolates (*p* = 0.0536; S2 Table).

*M*. *tuberculosis* isolates were classified into six families including Latin–American Mediterranean (LAM, n = 31, 29%), T (n = 23, 21.5%), Haarlem (H, n = 21, 19.6%), Ural (U, n = 6, 5.6%), S (n = 2, 1.9%) and USA (n = 1, 0.9%). The most prevalent spoligotype patterns were SIT 42/LAM 9 (n = 14, 13.1%), SIT 53/T1 (n = 8, 7.5%), and SIT 1800/T1 and SIT 50/H3, each with six isolates (5.6%).

## Discussion

The present study describes the genetic diversity of MTBC strains in humans and cattle from Northern Algeria. Regarding bovine TB, the *post-mortem* macroscopic inspection of bovine carcasses slaughtered in the four investigated abattoirs revealed a high prevalence (6.5%) of TB-like lesions compared to previously reported data from Algeria (3.6% in 2007) [15,16]. This value is also higher than the values reported in the neighboring countries, such as Morocco (3.7%) and Tunisia (3.2%) [25,26]; however, it is lower than the values reported in other African countries, such as Nigeria (9.3%) and Mali (11.9%) [27,28]. This variation might be explained by numerous parameters which are considered to be risk factors for bovine TB, such as age, gender, breed, immune status, etc. [29].

In the present study, we observed TB-like lesions in different organs including pulmonary and extra-pulmonary tissues. Nevertheless, over 95% (222/232) of noted lesions occurred in the lung tissue and/or lymph nodes. Among these, 30.2% were associated with extra-pulmonary organs and two-thirds (69.8%) were confined to the lungs, corroborating previous studies reporting that bovine TB mostly affects the lungs [25,27,30–32] as a result of the primary transmission route [33]. A minority of inspected carcasses (4.3%) presented generalized TB lesions. Generalized disease may occur if the initial immune response is ineffective or after reinfection [33].

In our study, 78% of bovine tissue samples submitted to culture were positive. It is worthy of note that although culture is considered to be the “gold standard” in TB diagnosis, its sensitivity is low and leaves much room for improvement [34]. In fact, its sensitivity varies according to the decontamination method and/or storage conditions of the samples [35]. In our study, due to logistic constraints, bovine samples were stored at -20°C for a maximum of five weeks prior culture processing, which may interfere with the viability of mycobacteria.

Based on spoligotyping, all cattle isolates were confirmed as MTBC, including *M*. *bovis*, *M*. *cap*rae, *M*. “*microti-like*” and *M*. “*pinnipedii-like*”, all of which are known for their potential contribution to zoonotic TB [13,36,37]. Interestingly, no isolates were attributed to non-tuberculous mycobacteria (NTM) species, which are often a cause of pulmonary diseases in cattle that resemble TB. The likely implication of NTM species in bovine TB was reported in Algeria with a proportion of 3% [15], as well as in numerous neighboring countries, such as Tunisia (3%), Mali (10.1%) and Niger (38.5%) [28,30,38].

The most abundant *M*. *bovis* spoligotypes observed in our study (SB0120, SB0121 and SB0134) are among the most common worldwide. SB0120 and SB0121 are the predominant *M*. *bovis* profiles circulating among animals including cattle, especially in Africa and continental European countries [39]. These two profiles are prevalent in Northern Africa, including in Algeria, Tunisia and Morocco, as well as in France, Spain, Italy, Portugal and Belgium [15,25,38,40–42]. The SB0134 spoligotype is frequently reported in Africa—for instance, in Algeria (7%), Tunisia (11.4%) and Mali (15%) [15,38,43]—and seems to be prevalent in France and Spain [40,41]. The importation of dairy cattle from Europe to North Africa, including Algeria—still common practice as of today—could explain the spread of these profiles. This hypothesis is consolidated by the phylogenetic link between *M*. *bovis* isolates from Algeria and France as reported by Sahraoui et al. [15]. Based on whole genome sequence analysis in a recent study comprising isolates from various geographical origins, SB0120 isolates were assigned to some new “unknown” clonal complexes (unknown 2, 3, 4 or 5), while all SB0121 isolates from the same study were grouped as belonging to the European 2 complex [44]. Some East-African SB0134 isolates were recently classified as part of the “unknown 2” clonal complex, while two European SB0134 were grouped under “unknown 7”. Among the less frequently known SB types, one was assigned to EU2 (SB0339, Germany), one as “unknown 2” (SB1003, unknown geographical origin), and some were grouped under “unknown 4” (SB0870 from Germany, SB0833, SB0860, SB0871 and SB0961 from unknown geographical origin) [44].

Regarding *M*. *caprae*, we identified two types (SB0835 and SB1451) from cattle in two slaughterhouses. *M*. *caprae* was previously isolated from bovine TB in Algeria and other African countries [25,26,38,45]. The infection of cattle by *M*. *caprae* could be explained by the cohabitation with goats [15]. In fact, the sharing of watering and grazing points between goats and cattle allows a close interspecies interaction among these domestic animals and therefore increases the likelihood of the cross-species transmission of mycobacteria [46–49].

It is particularly noteworthy that we identified seven new profiles from cattle, of which three presented with “atypical” profiles: SB2699 from the Sétif slaughterhouse grouping with SB2402, and from the El-Eulma slaughterhouse, profiles SB2700 and SB2701, which were closely grouped to *M*. *microti* and *M*. *pinnipedii*, respectively. Even though these were closely grouped by spoligotyping, our RD-PCR analysis could not confirm this “subspecies” identification. *M*. *microti* and *M*. *pinnipedii* have not been reported among cattle with TB-like lesions in Algeria to date. *M*. *microti* was originally identified as the cause of TB in wild rodents [50,51], but it can also infect other animals. It has been reported occasionally in cattle from France and South Africa [52,53]. *M*. *microti* can be transmitted directly from rodents to humans by domestic hosts such as cats, llamas, dogs or cattle [36,51]. *M*. *pinnipedii* causes TB in a number of pinniped species (marine mammals) worldwide [54]. Pinnipeds infected with *M*. *pinnipedii* in marine parks and zoos are the main source of infection for humans and other animals [54,55]. In New Zealand, the infection of cattle with *M*. *pinnipedii* was associated with contaminated water derived directly from the ocean or beach grazing areas where pinnipeds were found [56]. It remains unclear what the source of infection could be for these unusual MTBC strains in our area. Furthermore, additional genetic (whole genome sequencing) analysis is needed to further characterize these strains, as spoligotyping has limited discriminatory capacity, phylogenetic distances cannot be reliably determined [57] and the RD-PCR applied in this study targets only a limited number of RDs, not all of which are relevant for *M*. *bovis* typing [24,44].

Concerning human TB, we identified seven isolates as *M*. *bovis*. This is the first report of *M*. *bovis* from humans in Algeria. Infection with *M*. *bovis* in humans appears to occur mostly in developing countries, especially in Africa [58–63]. Most of our isolates were identified from pulmonary specimens, reflecting airway infection. This form of zoonotic TB occurs usually among people who have frequent contact with infected animals, such as farmers and veterinarians [64]. As for the extra-pulmonary forms, generally, the infection occurs by the consumption of unpasteurized dairy products from infected cows [65]. Dürr et al. [10] have suggested an association between zoonotic TB and extra-pulmonary infection, with predominant infections of the lymph nodes. In their study, Ghariani et al. [62] reported the occurrence of *M*. *bovis* in 76% of lymphadenitis cases. In the present investigation, patients with lymphadenitis TB were not included because the diagnostic of this form is only performed by the histopathological examination of lymph node biopsies. This therefore constitutes a bias when estimating the real implication of *M*. *bovis* in human cases. Another cause of the underestimation of *M*. *bovis* in human TB from our study may have been the fact that human samples were only grown on L–J medium, which contains glycerol, which partially inhibits the growth of *M*. *bovis* [66]. Nevertheless, in our study, *M*. *bovis* isolated from bovine tissues grew well on L–J medium, and so the culture medium bias for human samples may also have been limited.

As regards the zoonotic transmission of bovine TB in Northern Algeria, our data allow us to suggest a regional, causal link between bovine and human TB, similar to the previously reported positive correlations between the prevalence of bovine TB and human infection with *M*. *bovis* [11,12]. We observed a significantly higher prevalence of bovine carcasses with TB-like lesions in Sétif’s slaughterhouse (20%) as compared to the other slaughterhouses; on the other hand, being diagnosed at the Sétif hospital tended towards an association with *M*. *bovis* infection but did not reach significance. In addition, regarding bovine isolates, SB0120 was the most prevalent among human *M*. *bovis* isolates, while SB0121 and SB0860 were also identified. The observed higher bovine TB among cattle from Sétif might be linked to a difference in cattle population across the included slaughterhouses, with 40% of elderly females in Sétif compared to <1% in Béjaia. A more in-depth multivariate analysis including all risk factors is needed to further investigate this, which was beyond the scope of the current study. Furthermore, to document real chains of transmission, more in-depth genetic and epidemiological analysis over a longer period would be needed. In the current study, we did not have access to the socio-demographic data of patients (*e*.*g*., profession, consumer habits and HIV status) to assess the influence of these factors on human infection, especially on the zoonotic TB forms. Additionally, in current routine practice, details regarding the origin of animals are not registered in Algerian slaughterhouses, jeopardizing this investigation.

Our results demonstrated that majority of the 107 *M*. *tuberculosis* strains belonged to lineage 4—the most widespread lineage and among the more virulent globally [67]. The predominant clades identified in this lineage were LAM (29%), T (21.5%) and Haarlem (19.6%), which were reported in Algeria and in Northern African countries as large common clades [68]. The most prevalent sub-lineage LAM 9 (SIT 42, 13.1%) is predominant in North Africa as well as in a number European countries such as Spain, Italy and Belgium, sharing a close cluster with Northern Africa countries [69]. SIT 42 was previously reported in human TB in Algeria (19.3%) [70] and Morocco (24%) [71]. The second-most prevalent sub-lineage T1 (SIT 53, 7.5% and SIT 1800, 5.6%) is prevalent in 70% of African countries as well as in some European countries (France, Italy and Belgium) [69]. This sub-lineage seems to be implicated with a higher likelihood of antibiotic-resistance compared to other genotypes [72]. We do not have drug-susceptibility data for our isolates. While SIT 53 was previously reported in Algeria (25.3%) [70] and in other Africa countries such as Morocco (11.1%) [71], our study reports the presence of SIT 1800 in Algeria for the first time. The SIT 50/H 3 sub-lineage is typically implicated in human TB in African countries, such as in Algeria (19.3%) [70] and Morocco (5.3%) [71].

## Conclusion

The identification of a zoonotic mosaic MBTC species belonging to different geographical origins (Africa, Europe and Asia) would be of particular interest from the perspective of the current re-emergence/emergence of multidrug-resistant strains in Algeria. The possible implication of *M*. *bovis* in human TB in Northern Algeria cannot be ignored, since 7 out of 115 confirmed cases were due to this zoonotic species. We therefore conclude that a collaboration between the Ministry of Health and the Directorate of Veterinary Services in this country is crucial to further expand our understanding of *M*. *bovis* infection prevalence in cattle and its risks for human health. Furthermore, the establishment of traceability systems for livestock from the farm to slaughterhouse is an urgent necessity. This kind of device would allow the generation of data and relevant information on the life of an animal since birth until the end of its life to be collected. Thus, traceability systems would limit the spread of infectious agents (e.g., *M*. *bovis*, *Brucella* spp., etc.) in the herd as well as from animals to humans by acting upstream.

## Supporting information

S1 Fig Phylogenetic tree of *Mycobacterium tuberculosis* complex (MTBC) genotypes (n = 97 spoligotype patterns) from humans and cattle in two departments of Northern Algeria (2017–2019), using the MIRU-VNTR*plus* international database (). (TIF)

S2 Fig Tuberculosis lesions associated with *M*. “*microti-like*” and *M*. “*pinnipedii-like*” infection in cattle. (**A)** Caseous-calcified lesion of the right tracheobronchial lymph node by *M*. “*microti-like*” (SB2700), miliary TB infection by *M*. “*pinnipedii-like*” (SB2699) in lungs and liver **(B)**, diaphragm **(C)**, digestive tract **(D)** and spleen **(E)**.
(TIF)

S1 Table Overview of 43 *Mycobacterium bovis* and related spoligotypes identified in human and bovine samples in the present study, stratified according to previous reporting in Algeria or neighboring North African and South European countries. (DOCX)

S2 Table The distribution of human MTBC species did not differ significantly according to the disease presentation or hospital district. (DOCX)

S1 Text Protocol: Lineage-specific single-nucleotide polymorphisms (SNPs) used in this study for genotyping of *Mycobacterium tuberculosis* complex isolates. (PDF)
