# Supplementary figures and images for: Broad diversity of Mycobacterium tuberculosis complex strains isolated from humans and cattle in Northern Algeria suggests a zoonotic transmission cycle
Source: PLoS Negl Trop Dis. 2020 Nov 30;14(11):e0008894. doi: 10.1371/journal.pntd.0008894 (PMC7728391; doi:10.1371/journal.pntd.0008894)

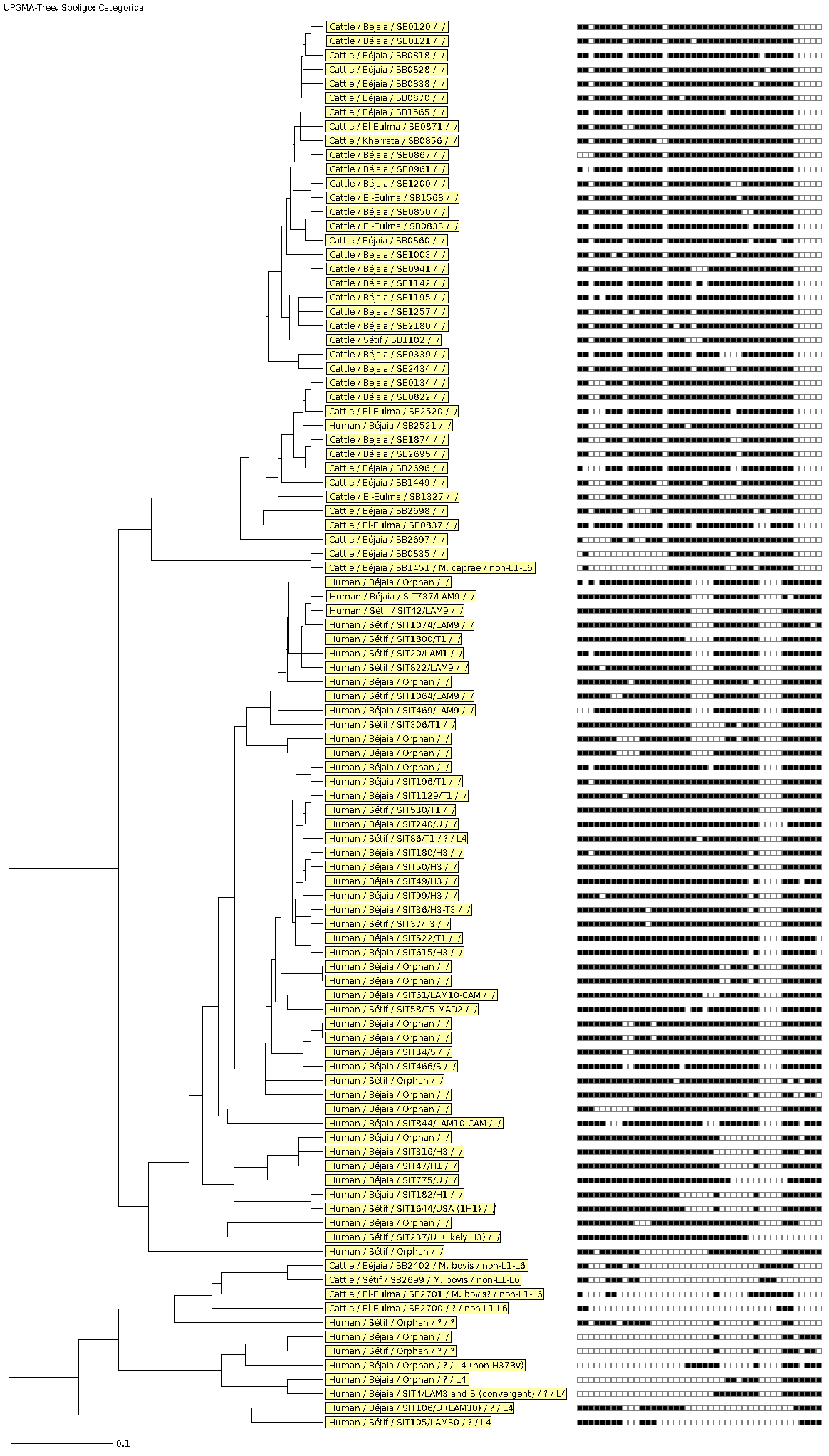

Supplement: S1 Fig — (TIF) [file pntd.0008894.s001.tif]

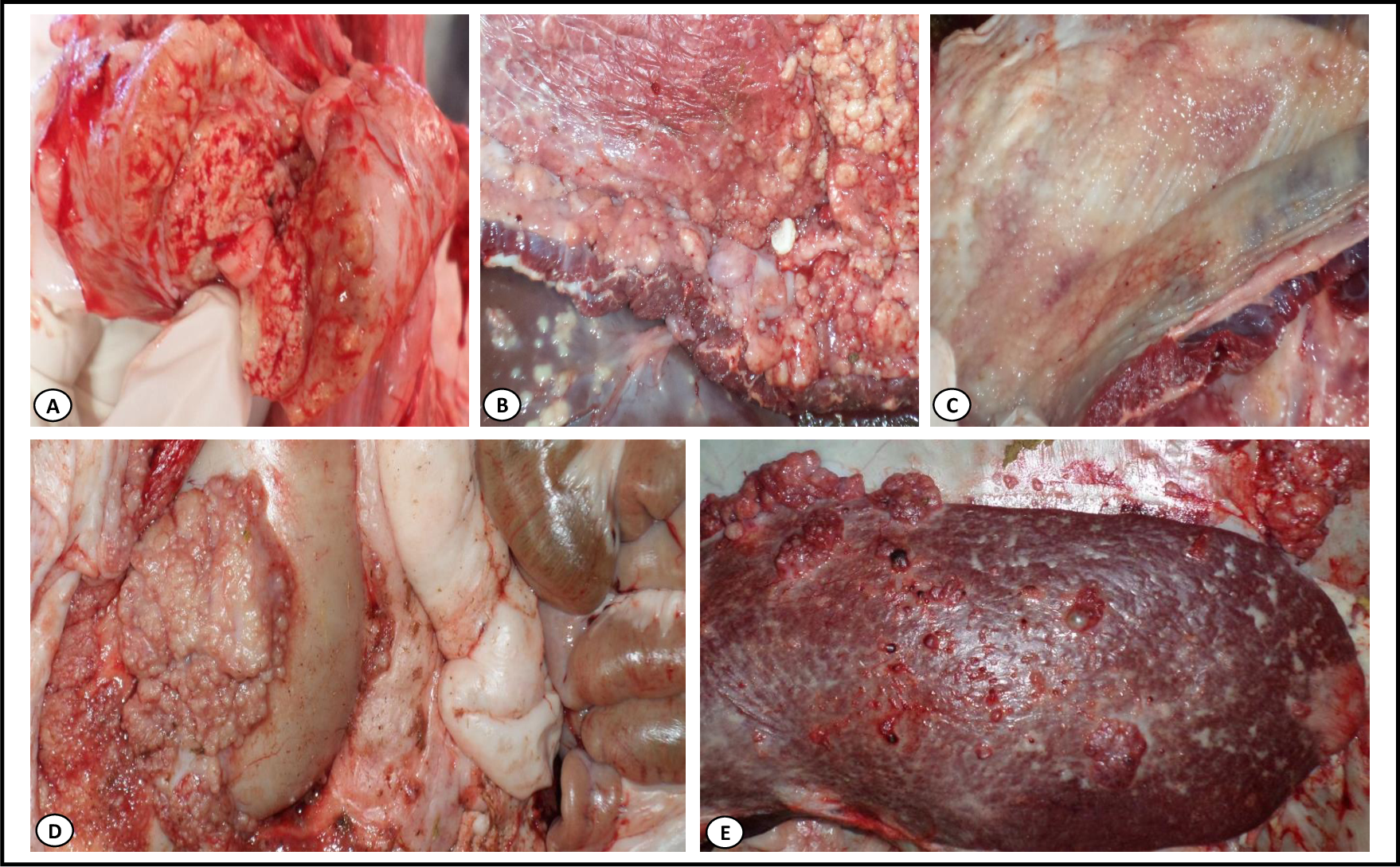

Supplement: S2 Fig — (A) Caseous-calcified lesion of the right tracheobronchial lymph node by M. “microti-like” (SB2700), miliary TB infection by M. “pinnipedii-like” (SB2699) in lungs and liver (B), diaphragm (C), digestive tract (D) and spleen (E). (TIF) [file pntd.0008894.s002.tif]
