# Supplementary material for: Broad diversity of Mycobacterium tuberculosis complex strains isolated from humans and cattle in Northern Algeria suggests a zoonotic transmission cycle
Source: PLoS Negl Trop Dis. 2020 Nov 30;14(11):e0008894. doi: 10.1371/journal.pntd.0008894 (PMC7728391; doi:10.1371/journal.pntd.0008894)
Supplement: S1 Table — (DOCX) [file pntd.0008894.s003.docx]

**S1 Table**. Overview of 43 *Mycobacterium bovis* and related spoligotypes identified in human and bovine samples in the present study, stratified according to previous reporting in Algeria or neighboring North African and South European countries.

| Spoligotype  profiles identified in the present study | Previously reported  in Algeria* [15] | Previously reported in North African and South European countries | | | | | | |
| --- | --- | --- | --- | --- | --- | --- | --- | --- |
|  |  | **Tunisia** [26,38] | **Morocco** [25] | **Chad*** | **Mali** [43] | **Italy**  [73,74] | **Spain**  [75] | **France*** [40] |
| SB0120^$^, SB0121^$^  SB0134, SB0822  SB0828, SB0837  SB0850, SB0860^$^  SB0867, SB0941  SB1200, SB1449  SB2520, SB2521^£^  SB1451 *M. caprae* | Yes | SB0120  SB0121  SB0134  SB0828  SB1200 | SB0120  SB0121  SB0134 | No | SB0134 | SB0120  SB0121  SB0134  SB0822  SB0828  SB0850  SB0867  SB1200 | SB0120  SB0121  SB0134  SB0822  SB0828  SB0850  SB0860 | SB0120  SB0121  SB0134  SB0822  SB0828  SB0837  SB0850  SB0860  SB0867  SB0941 |
| SB0339, SB0818, SB0833 SB0838, SB0856, SB0870 SB0871, SB0961, SB1003 SB1102, SB1142, SB1195 SB1257, SB1327, SB1565 SB1568, SB1874, SB2180 SB2402, SB2434, SB2695 SB2696, SB2697, SB2698 SB2699  SB0835 *M. caprae*  SB2700 *M. microti-*like  SB2701 *M. pinnipedii*-like | No | SB0856  SB1003  SB0871 | SB0339  SB0856 | SB1102 | No | SB0818  SB0833  SB0838  SB0961  SB1565  SB1568 | SB0339  SB0818  SB0833  SB0856 SB1195  SB1257  SB1327 SB1874  SB2180  SB2434 | SB0818  SB0833  SB0838  SB0856  SB0870  SB0871  SB2402  SB0835 |

* Spoligotype registered in “The *Mycobacterium bovis* Spoligotype”. **^$^** SB types have been isolated from both bovine and human samples in the present study; ^£^ SB type has been isolated from one human sample only; SB types marked in bold have not been reported in neighboring or South European countries; Underlined SB types belonged to a spoligotype cluster in the present study.

73. Boniotti MB, Goria M, Loda D, Garrone A, Benedetto A, Mondo A, et al. Molecular typing of *Mycobacterium bovis* strains isolated in Italy from 2000 to 2006 and evaluation of variable-number tandem repeats for geographically optimized genotyping. Journal of clinical microbiology. 2009;47: 636–44. doi:10.1128/JCM.01192-08

74. Marianelli C, Amato B, Boniotti MB, Vitale M, Pruiti Ciarello F, Pacciarini ML, et al. Genotype diversity and distribution of *Mycobacterium bovis* from livestock in a small, high-risk area in northeastern Sicily, Italy. PLoS neglected tropical diseases. 2019;13: e0007546. doi:10.1371/journal.pntd.0007546

75. Rodríguez Campos S. Molecular epidemiology of *Mycobacterium bovis* and *Mycobacterium caprae* in Spain. The Complutense University of Madrid. 2012. Available: https://www.google.com/url?sa=t&rct=j&q=&esrc=s&source=web&cd=&cad=rja&uact=8&ved=2ahUKEwjQ9ITAq83pAhUQxoUKHRjNCywQFjABegQIBBAB&url=https%3A%2F%2Fwww.visavet.es%2Fdata%2Ftesis%2Fmolecular-epidemiology-of-*mycobacterium*-*bovis*-and-*mycobacterium*-*caprae*-in-spain.pdf&usg=AOvVaw1r0hR-D8DMx3c062fsb5sU
