# Supplementary material for: Broad diversity of Mycobacterium tuberculosis complex strains isolated from humans and cattle in Northern Algeria suggests a zoonotic transmission cycle
Source: PLoS Negl Trop Dis. 2020 Nov 30;14(11):e0008894. doi: 10.1371/journal.pntd.0008894 (PMC7728391; doi:10.1371/journal.pntd.0008894)
Supplement: S2 Table — (DOCX) [file pntd.0008894.s004.docx]

**S2 Table.** The distribution of human MTBC species did not differ significantly according to the disease presentation or hospital district.

|  | *Mycobacterium tuberculosis* | *Mycobacterium bovis* | *Odds ratio; p* |
| --- | --- | --- | --- |
| Pulmonary | 78 | 5 | OR 10.40 (95% CI 0.19-5.65); *p* = 0.9638 |
| Extra-pulmonary | 30 | 2 |  |
| Sétif | 24 | 4 | OR 46.67 (95% CI 0.98-22.30); *p* = 0.0536 |
| Béjaia | 84 | 3 |  |
