# Supplementary material for: Broad diversity of Mycobacterium tuberculosis complex strains isolated from humans and cattle in Northern Algeria suggests a zoonotic transmission cycle
Source: PLoS Negl Trop Dis. 2020 Nov 30;14(11):e0008894. doi: 10.1371/journal.pntd.0008894 (PMC7728391; doi:10.1371/journal.pntd.0008894)
Supplement: S1 Text — (PDF) [file pntd.0008894.s005.pdf]

Supporting Methods

PCR of L125, L346 PCR for SNP based lineage detection: Amplification program, primers and SNP loci from Inaki's group

Table 1. Amplification program

Step	Temperature	Time
Initial denaturation	95°C	3 min
Denaturation	95°C	30 s
Annealing	57°C (Not ok in ITM)	30 s
Extension	72°C	30 s
Final extension	72°C	10 min

Table 2. Primers used for Sanger sequencing amplification

MTBC lineage	SNP position	Nucleotide change	Genre	Primers 5'-3'	Amplicon size (bp)
Lineages 1, 2, 5	L1 4357773	G/A	Rv3878/Rv3879c	F-ACCCTCAACAACCACAACGT<br>R-CGACACTACCGATCAGCGTT	386
	L2 4357804	T/G			
	L5 4357657	G/A			
Lineages 3, 4, 6	L3 1281984	G/A	Rv1155/intergenic region	F-GATGGTCATACGCCGTTGCT<br>R-CTCTTGCGGGGACTTCGATT	402
	L4 1281771	C/T			
	L6 1281685	C/G			

bp: base pairs

Table 3. Mix preparation for PCR (isolates)

PCR mix ("modified ITM's 25 pmol/50µL reaction")	L125 PCR	L346 PCR
	**For 20 reactions (µL)**	**For 20 reactions**
Water (for 10 µL DNA reaction)	180	Use water in replacement of Q solution (180 µL)
Buffer 2x	500	500
dNTP (2.5 mM)	80	80
Primer 1 (25pmol/µL)	20	L346 primer 1 (20 µL)
Primer 2 (25pmol/µL)	20	L346 primer 2 (20 µL)
Taq 5U/µL	4	4
Distribute 40 µL per tube<br>Add 10 µL DNA per tube		

Table 4. Amplification program for isolates (L125 and L346 PCRs)

Step	Temperature	Time
Initial denaturation	95°C	3 min
Denaturation	95°C	30 s
Annealing	67.4°C (Ok)	30 s
Extension	72°C	30 s
Final extension	72°C	10 min
Number of cycles	**45**	

Table 5: Mix preparation for PCR (sputum)

PCR mix (“modified ITM’s 25 pmol/50µl reaction”)	L125 PCR	L346 PCR
	**For 20 reactions (µL)**	**For 20 reactions**
Q solution (for 10 µL DNA reaction)	180	Use water in replacement of Q solution (180 µL)
Buffer 2x	500	500
dNTP (2.5 mM)	80	80
Primer 1 (25pmol/µL)	20	L346 primer 1 (20 µL)
Primer 2 (25pmol/µL)	20	L346 primer 2 (20 µL)
Taq 5U/µL	4	4
Distribute 40 µL per tube<br>Add 10 µL DNA per tube		

Table 6: Amplification program for sputum

Step	Temperature		Time
	L125 PCR (sputum)	L346 PCR (sputum)	
Initial denaturation	95°C	95°C	3 min
Denaturation	95°C	95°C	30 s
Annealing	64.9°C (OK)	69.3°C (Ok)	30 s
Extension	72°C	72°C	30 s
Final extension	72°C	72°C	10 min
Number of cycles	**60**		

Use as positive PCR control a DNA extract from reference strain

Either L1, L2, L5 strain for the L125 PCR

Either L3, L4, L6 strain for the L346 PCR

Table 7: Examples of reference strains used as positive controls

ID	Lineage
18-00082	L1
18-00083	L1
18-00085	L2
18-00086	L2+
18-00087	L2
18-00089	L3
18-00090	L3
18-00093	L4
18-00094	L4
18-00095	L5
18-00096	L5
18-00098	L6
18-00099	L6

Sequence analysis (see table 1 above for expected SNP and loci per lineage)

- To analyze genome sequence after L125 sequencing, use the reference H37Rv isolate or L4 isolate (180093) sequence (L125) as reference genome for alignment. For sequence from L125 PCR, all SNP loci (from L5, L1, L2) were well covered. Sense and reverse sequences were analyzed together.
- To analyze genome sequence after L346 sequencing, use the reference L5 isolate (180095) or L2 isolate (180085) sequence (L346) as reference genome for the alignment. For sequence from PCR L346, Primer sense need to start about 60 bases backward compared to now. Sense and reverse sequences were analyzed separately in order to interpret all the 3 SNP loci (L6, L4, L3).

  Reverse primer covered all the 3 loci to analyze. But the last locus on the sense and reverse complement of reverse strands (L3) is almost 17 nucleotides from the poorly aligned ending part of the sequence.
- Even when a SNP is not found on the DNA sequence, based on the spoligotype pattern and expected lineage mixture, check for double pic on the sequences. Eg. In two specimens (suspected mixed infection L2+L4 based on spoligotype pattern), only the SNP for L4 was found on the AND sequence. But, when we looked at the curve, there

was a double pic at the locus for L2 SNP. The double pic included the wild-type nucleotide pic and the L2 SNP nucleotide pic.

Results on lineage reference isolates

After sequencing, expected results were obtained for all lineage reference isolates.

Results on sputum (DNA extracted from smear-positive sputa using Maxwell extraction method)

When at the optimal annealing temperature found, the PCR was negative, positive with a weak band, or with non-specific band, the PCR was repeated with the next lower temperature of the gradient range (as annealing temperature).
